# Shoulder proprioception – lessons we learned from idiopathic frozen shoulder

**DOI:** 10.1186/s12891-016-0971-5

**Published:** 2016-03-12

**Authors:** Jaroslaw Fabis, Remigiusz Rzepka, Anna Fabis, Jacek Zwierzchowski, Grzegorz Kubiak, Arkadiusz Stanula, Michal Polguj, Radek Maciej

**Affiliations:** Department of Arthroscopy Minimally Invasive Surgery and Sport Traumatology, Medical University of Lodz and FMC Medical Centre Lodz, Lodz, Poland

**Keywords:** Frozen shoulder, Isokinetics, Proprioception, Passive stabilizers

## Abstract

**Background:**

Of all the most frequent soft tissue disorders of the shoulder, idiopathic frozen shoulder (IFS) offers the greatest potential for studying proprioception. Studies concerning the presence of proprioception dysfunctions have failed to determine the potential for spontaneous healing of passive shoulder stabilizers (anterior and posterior capsule, middle and inferior gleno-humeral ligaments), its relationship with passive (PJPS) and active (AJPS) shoulder proprioception for internal and external rotation (IR, ER), as well as the isokinetic muscle performance of the internal and external rotators. This study investigates these dependencies in the case of arthroscopic release of IFS.

**Methods:**

The study group comprised 23 patients (average aged 54.2) who underwent arthroscopic release due to IFS and 20 healthy volunteers. The average follow-up time was 29.2 months. The Biodex system was used for proprioception measurement in a modified neutral arm position and isokinetic evaluation. The results were analysed using the T-test, Wilcoxon and interclass correlation coefficient. *P*-values lower than 0.05 were considered significant.

**Results:**

Statistically significant differences were found between involved (I) and uninvolved (U) shoulders only in the cases of PJPS and AJPS, peak torque, time to peak torque and acceleration time for ER (*p* < 0.05). No statistically significant difference was noted between PJPS IR and PJPS ER or between AJPS IR and AJPS ER (*p* > 0.05) for the U shoulders.

**Conclusions:**

The anatomical structure of anterior (capsule, middle and anterior band of inferior gleno-humeral ligament) and posterior (capsule and posterior band of inferior gleno-humeral ligament) passive shoulder restraints has no impact on the difference in PJPS values between ER and IR in a modified neutral shoulder position. The potential for spontaneous healing of the anterior and posterior passive shoulder restraints influences PJPS recovery after arthroscopic release of IFS. ER peak torque deficits negatively affect AJPS values. PJPS and AJPS of ER and IR can be measured with a high level of reproducibility using an isokinetic dynamometer with the arm in a modified neutral shoulder position. Differences greater than 15 % for PJPS and >24 % for AJPS for ER and IR can be helpful for future studies as baseline data for identification of particular passive and active shoulder stabilizers at risk.

## Background

The most frequently studied forms of shoulder joint proprioception are passive and active joint position sense (PJPS and AJPS) [1–10]. However, knowledge regarding proprioception dysfunctions remains incomplete, and the potential for passive shoulder stabilizers to spontaneously heal, as well as the relationship between the healing process and the position senses, is not fully understood [1, 3, 4, 6, 7,]. Hence, the precise relationship between the anatomical structure of the anterior and posterior passive shoulder stabilizers and PJPS or AJPS remains unclear, and relationship between them and the isokinetic muscle performance demands further clarification. Similarly, previous studies have been unable to reach consensus on the optimal position and equipment which should be used for measurement and normative PJPS and AJPS values [1–10].

A recent literature review reveals a lack of research concerning the evaluation of PJPS and AJPS after arthroscopic capsule-ligamentotomy for idiopathic frozen shoulder (IFS), despite it being one of the most common disorders of the soft tissues of the shoulder joint [11]. Selective release of the anterior-inferior-posterior joint capsule, medial gleno-humeral ligament (MGHL) and inferior gleno-humeral ligament (IGHL) has many advantages: not only is it conscious, precise and reproducible, it is an effective way of treating certain cases and gives positive results [12–17]. Hence, IFS offers great potential for the study of PJPS and AJPS and the relationship between them.

Assuming that the difference between the anatomical structure of the anterior and posterior passive shoulder stabilizers has an impact on proprioception and its spontaneous recovery after arthroscopic release of idiopathic frozen shoulder, the aims of this study were as follows: 1) to evaluate the influence of the anterior and posterior capsule (AC and PC), the middle gleno-humeral ligament (MGHL) and the anterior and posterior bands of inferior gleno-humeral ligament (ABIGHL and PIGHL) on the PJPS and AJPS values for internal and external rotation (IR, ER) after arthroscopic release, with regard to the isokinetic performance of the shoulder rotators; 2) to evaluate the reproducibility and clinical value of measuring proprioception under minimal stimulation of proprioreceptors thanks to the modified neutral position of the arm (MNP) [18] with the use of an isokinetic dynamometer; 3) to create a baseline data of normative PJPS and AJPS values for IR and ER for future studies.

## Methods

The study group included 23 patients (16 female and 7 male) aged 54.2 (range 37–67) of 27 [16] who underwent arthroscopic capsule-ligamentotomy due to idiopathic frozen shoulder (IFS). The average follow up time was 29.2 months (range, 26–47.3). The operation was performed at least 6 months after non-surgical treatment, which had demonstrated no improvement. The surgical procedure involved limited antero-posterior synovectomy (ablator Linvatec), interval resection and antero-inferior capsule resection, together with both MGHL and IGHL and posterior capsule resection with punch [16], The procedure was conducted by one surgeon (first author). Rehabilitation, comprising both passive and active exercises, was initiated soon after the patient regained consciousness, beginning the first post-operative day. All the patients were subjected to the same rehabilitation protocol mode: continuous passive motion device exercises (2 × 30 min) scapula and shoulder mobilization, as well as isometric and isotonic exercises of the shoulder abductors, external/internal rotators and the shoulder itself. At home, the patients performed stretching exercises and isometric and isotonic exercises of the aforementioned muscles three times a day for 20–30 min. No abduction splints were used.

The measurement protocol was approved by the Medical University of Lodz Bioethics Committee (RNN/193/12/KB). The patients who met the study inclusion criteria were familiarized with the study protocol and gave their written consent to the study before taking part.

The following study group inclusion criteria were adopted: the patient was at last 2 years from arthroscopic release; the patient had undergone a unilateral capsule-ligamentotomy procedure due to idiopathic frozen shoulder in the stage 2 (sever limitation of motion combine with some relief of pain) ((limitation of all shoulder motions, negative x-ray and sonographic evaluation); a negative history of diabetes and previous injuries for both the operated and healthy shoulder; an absence of shoulder pain (involved and contralateral) and neurological deficits of upper extremities at the time of the measurement; more than 90 % of anterior flexion, internal and external rotation present at 90° abduction in the scapular plane; the patient was able to undergo the intended measurements.

A Biodex System 3 isokinetic dynamometer (Biodex, Inc, USA) was used to measure all proprioception components and muscle performance. Prior to the measurement, the system was calibrated according to the instructions and recommendations of the producer. Before the measurement, each patient was given a thorough explanation of the study methodology and instructed as to the accuracy of the measure and the mode of communication with the researcher. The APJS and PJPS values of the gleno-humeral joint of the patients who met the given study inclusion criteria were measured on both the uninvolved (U) and involved (I) sides during IR and ER. When completing the measurement protocol, the U limb was tested first. The measurement was repeated 3 times and the obtained values were averaged and subjected to statistical analysis. Additionally, intraclass correlation coefficients (ICC) were used to determine the test-retest reliability of proprioception measurement in 20 randomly-selected healthy volunteers (10 male and 10 female; average age 24.5 years, range 18–38). All underwent PJPS and AJPS evaluation with two investigators who had been trained in the same protocol evaluation. Each subject completed a questionnaire regarding medical history to rule out subjects with neuromuscular or musculoskeletal injuries. Subject selection criteria included no history of upper extremity pathology or injury, a range of motion with a similar extent as the U side in the study group, as well as negative neurological and sonographic evaluations of the shoulders. Each subject was required to sign an informed consent form. Two test sessions were scheduled 4 days apart and were carried out at approximately the same time of day to ensure consistent activity levels.

Proprioception measurements were carried out with patients seated. To limit visual and acoustic stimuli during the procedure, bands were placed over the eyes of the patients and ear plugs were inserted. The patients were also stabilized with shoulder (both right and left) and hip straps fastened to the chair. To limit sensory stimuli from the skin during the proprioception test, the forearm in contact with the dynamometer was placed in an air splint (URIAS splint, Johnstone, 40–50 cm long). During both the AJPS and PJPS tests, the patient held a remote control which could be used to stop the dynamometer in the required position.

**Fig. 1:**
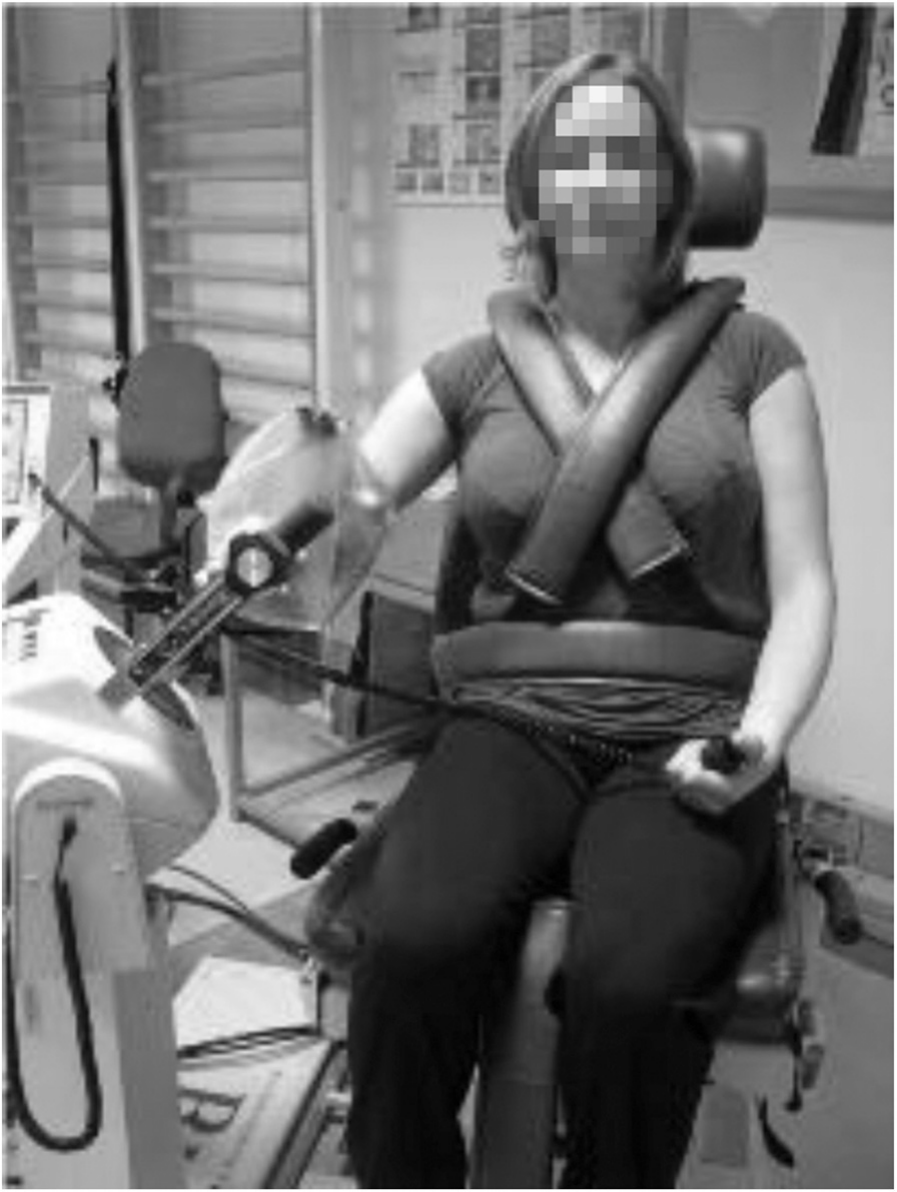
Modified neutral shoulder position

The proprioception measurements on the dynamometer were carried out in the MNP: the dynamometer was tilted 30° from horizontal base position, and the gleno-humeral joint placed at 30° of abduction and 30° of forward flexion into the plane of the scapula [18] (Fig. 1).

**Fig. 2:**
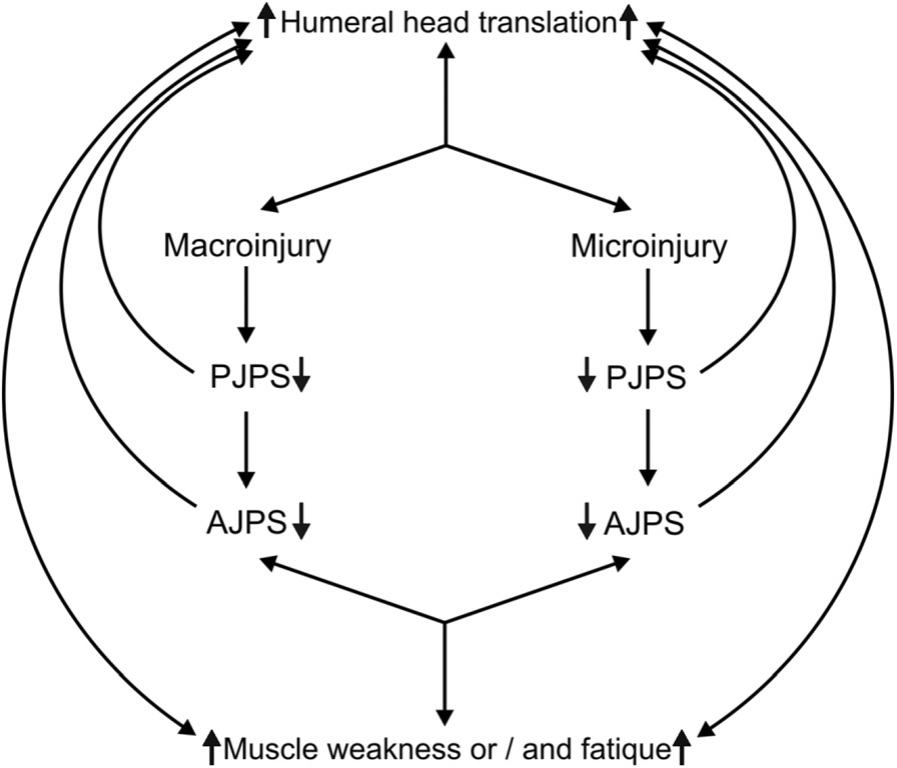
The concept of the vicious circle of the dependencies between passive and active joint position sense (PJPS and AJPS), macro- and micro-injury of passive stabilizers and muscle performance

### The active and passive joint position senses of gleno-humeral joint measurement

For AJPS evaluation at 30° external and internal rotation, the time between trials for external and internal rotation was 60 s. Before each trial, the patient was presented with a position which had to be actively imitated. The time to memorize the position was 10 s. After reaching the required joint position, the patient pressed the button to block the dynamometer. For PJPS evaluation at 30° ER and IR, the protocol was similar but the dynamometer arm initiated the motion in the given direction from the initial position at a constant angular velocity of 1° /s.

### Isokinetic evaluation of internal and external rotation

Isokinetic evaluation of ER and IR was performed in the MNP with 180° speed [18]. The peak torque, average peak torque, time to peak torque, acceleration and deceleration times were measured and then used for further statistical evaluation.

### Statistical analysis

The arithmetic mean and standard deviation were calculated from basic position measurements. The Shapiro-Wilk test was used to test the distribution of the data. For mean values with normal distributions, the parametric Student's t-test for dependent samples was used to identify statistically significant differences between the operated (I) and unoperated (U) limbs. For non-normal variables, the non-parametric rank-sum Wilcoxon test was implemented. Additionally, the Intraclass Correlation Coefficient (ICC) was used to evaluate the test-retest reliability of the measurements. A significance level of *p* < 0.05 was accepted. All the calculations were performed with STATISTICA ver.10 (StatSoft, Inc. 2011).

## Results

Statistically significant differences were found between the I and U shoulders only in the cases of PJPS and AJPS for ER (Table 1). The isokinetic evaluation revealed statistically significant differences between controls and the operated shoulders regarding peak torque, average peak torque, time to peak torque and acceleration time for ER and time to peak torque for IR (Table 1). No statistically significant differences were noted between PJPS IR and PJPS ER (*p* = 0.738) or between AJPS IR and AJPS ER (*p* = 0.132) for the U shoulder (Table 1).

**Table 1:** Basic data of active and passive joint position senses (AJPS, PJPS) for internal and external rotation (IR and ER) and isokinetic parameters for 180° speed for the involved (I) and uninvolved (U) shoulders of 23 patients operated on for idiopathic frozen shoulder, with statistical data regarding the particular comparisons between them (Wilcoxon test; *p* <0.05)

Parameters	Shoulder	Mean	SD	Range	*p* Value
AJPS IR ^0^	I	5.03	3.53	1.3–15.3	0.054
	U	3.84	1.97	2.0–10.3	
AJPS ER^0^	I	6.56	3.52	2.0–14.3	0.013
	U	4.71	2.50	1.7–11	
PJPS IR^0^	I	4.23	1.41	2.3–7.3	0.112
	U	3.59	1.44	1.7–6.0	
PJPS ER^0^	I	5.37	2.48	2.0–12.3	0.024
	U	3.80	1.82	1.3–7.3	
Peak Torque IR (Nm)	I	16.60	11.98	3.0–52.9	0.187
	U	18.30	11.37	3.2–46.6	
Peak Torque ER (Nm)	I	13.76	8.67	2.2–34.6	0.011
	U	15.82	7.09	6.8–32.8	
Average Peak Torque IR (Nm)	I	14.60	11.76	2.5–51.3	0.119
	U	16.35	10.66	2.2–41.3	
Average Peak Torque ER (Nm)	I	12.44	8.31	1.2–32.0	0.015
	U	14.27	7.04	4.8–32.2	
Time to Peak Torque IR (msec)	I	432.17	151.81	230–800	0.003
	U	364.78	117.20	210–650	
Time to Peak Torque ER (msec)	I	423.91	237.04	180–1120	0.012
	U	332.61	110.83	200–680	
Acceleration time IR (msec)	I	256.52	96.18	100–490	0.196
	U	250.44	109.77	100–530	
Acceleration time ER (msec)	I	286.52	118.19	100–560	0.007
	U	230	74.53	110–380	
Deceleration time IR (msec)	I	335.23	133.07	130–650	0.224
	U	301.74	138.36	130–600	
Deceleration time ER (msec)	I	280	120.30	130–590	0.284
	U	251.3	107.51	100–480	

The interclass correlation coefficients (ICC) of 40 shoulders of 20 healthy volunteers confirm that using a Biodex dynamometer to measure PJPS and AJPS with the arm in the MNP allows proprioception to be assessed with high reliability (Table 2).

**Table 2:** The average values, standard deviation (SD) and range of active and passive joint position sense (AJPS and PJPS) for internal and external rotation (IR and ER) of 40 shoulders from 20 healthy volunteers, together with the test retest evaluation of the inter-observer correlation coefficient (ICC) between 2 measurements

	Measurement (^0^)		ICC
	1	2	
AJPS IR	2.94 (1.25), 1.0–5.8	3.00 (1.11), 1.0–5.2	0.97
AJPS ER	2.87 (1.33), 0.9–6.1	2.89 (1.09), 1.2–5.2	0.95
PJPS IR	2.40 (1.34), 0.3–5.3	2.64 (1.16), 1.0–5.0	0.96
PJPS ER	2.39 (1.38), 0.3–5.3	2.55 (1.26), 0.4–5.1	0.96

The relationship between two consecutive measurements of AJPS and PJPS, for both IR and ER, for the healthy volunteers was not significant. Similarly, the comparison between the average values of IR PJPS and ER PJPS was insignificant, as was the relationship between the average values of IR AJPS and ER AJPS (Table 3).

**Table 3:** The comparison between two consecutive measurements of active and passive joint position sense (AJPS and PJPS) for internal and external rotation (IR and ER) of 40 shoulders within the group of 20 healthy volunteers (Wilcoxon test: *p* was significant at < 0.05)

Parameters	*p* Value
AJPS 1st measurement IR/ER	0.73
AJPS 2nd measurement IR/ER	0.45
PJPS 1st measurement IR/ER	0.95
PJPS 2nd measurement IR/ER	0.51
Average AJPS IR/ER	0.6
Average PJPS IR/ER	0.58

## Discussion

This is the first study to confirm that the anatomical structure of the anterior (capsule, MGHL, ABIGHL) and posterior (capsule and PBIGHL) passive shoulder restraints has no impact on the range of PJPS for either ER or IR, and that their potential for spontaneous healing affects the recovery of proprioception after arthroscopic release of idiopathic frozen shoulder. Previous studies of shoulder proprioception have used various sets of equipment and a range of arm positions [1–10, 19–24]. One of the devices used to study proprioception is the isokinetic dynamometer [1, 6, 10, 20], which allows measurement of the peak torque of the muscle responsible for shoulder stability and injury prevention [18, 24,].

Shoulder position plays a crucial role in interpreting the results of PJPS and AJPS assessment. Since more tension is created in the passive and active restraints, and thus the tension of their respective mechanoreceptors, Golgi organs and muscle spindles, at the terminal points of the range of motion [25–28], this influences the assessment of PJPS and AJPS. Massimini et al. [27] note that the elongation of the MGHL, the anterior band of the IGHL (ABIGHL) and the posterior band of the IGHL (PBIGHL) are less at 45° of abduction than at 90° of abduction and at 90° of abduction combined with ER and IR. Thus, placing the arm in the MNP allows relatively minimal tension to be placed on particular passive shoulder restraints. This, together with the fact that the isokinetic dynamometer provides stable and precise arm support, combined with reduction of rotator cuff and scapular muscle tension [18] the MNP offers very good sensitivity for measuring PJPS and AJPS for ER and IR. Moreover, the MNP is also very close to 45° of abduction in the scapular plane, which has been demonstrated to facilitate reliable isokinetic assessment of shoulder IR and ER strength [29, 30].

One unexpected finding of our study was the lack of statistically significant differences between the PJPS values measured for ER and IR, both for U shoulders and within the group of healthy volunteers. This indicates that anatomical differences between anterior and posterior passive stabilizers, and differences in the distribution of the particular types of mechanoreceptors contained therein [31–35], do not affect PJPS in MNP.

Our study is the first to reveal the spontaneous ability of the PC and PBIGHL to heal and recover sufficient tension for normalization of IR PJPS post-capsuloligamentotomy in idiopathic frozen shoulder. However, in the case of ER rotation, the more complex anatomical structure and wider area of insertion of the MGHL and ABIGHL, in contrast to PBIGHL [33], did not allow sufficient spontaneous healing to take place and for PJPS to be normalized. These findings also support earlier data indicating that the capsule mechanoreceptors influences shoulder proprioception [31, 34, 35].

The results of isokinetic testing are even more convincing (Table 1). The isokinetic test and results of AJPS and PJPS evaluation strongly suggest that besides the impairment of muscle peak torque and time to peak torque, AJPS was also dependent on afferent information from mechanoreceptors of the passive stabilizers while in the MNP (Table 1). In particular, no statistically significant difference was found between I and U with regard to deceleration time for ER (Table 1). Hence, a thorough evaluation of the passive stabilizers should be performed in the case of AJPS impairment [36].

These observations have particular clinical significance. In the case of passive stabilizer insufficiency, the “stability over mobility” mechanism is activated [37]. Although this mechanism allows greater control over shoulder stability, it can impair the function of the shoulder further by influencing the neuromuscular control of agonists and antagonists [37–42]. Wuelker et al. [43] report a 46 % increase of anterior humeral head displacement and 31 % increase of posterior humeral head displacement when rotator cuff forces are reduced by 50 %, and von Eisenhart-Rothe et al. [44] confirm the importance of arm position and muscle activity for gleno-humeral translation in patients with traumatic shoulder instability. Therefore, even subtle injury of the passive stabilizers may influence the PJPS and ultimately, shoulder stability, especially in case of decreased muscle peak torque [40–45]. Furthermore, as muscle fatigue decreases the peak torque and the AJPS value of the shoulder [24, 45], the tensile stress placed on the passive restraints during overhead activities further increases. Therefore, our findings support those of earlier studies, which indicate that, together with careful clinical and proprioception examination, isokinetic testing should be a part of any global shoulder function evaluation in overhead sport activities [18, 24, 38, 46]. Figure 2 summarizes the author’s concept of the vicious circle of the dependencies between PJPS and AJPS, passive stabilizers, macro- and micro-injury of passive stabilizers and muscle performance.

The present study has some limitations. The age of the patients is one factor, as proprioception is known to deteriorate with age [47]. However, as the deterioration of proprioception is the result of similar structural and functional changes within both anterior and posterior shoulder passive stabilizers, nervous system and the muscles employed for IR and ER, its decline should not result in significant differences in PJPS and AJPS between the control and operated shoulders examined in the present study. Changes in the passive restraints caused by the inflammatory nature of idiopathic frozen shoulder may also influence the results. As the presence of synovitis prevented any determination of the degree of formation of the MGHL in some cases, no such assessment was included in the study. As the MGHL is somewhat visible in 42 %, distinct in 49 % and clearly visible in 9 % of cases, based on the classification of Gohlke et al. [48], the structure was always released, regardless of its variant of formation.

As the measurement of proprioception in the proposed MNP using an isokinetic dynamometer was found to have very good reliability, it may serve as a standard means of identifying PJPS and AJPS disorders in shoulder IR and ER in overhead sports activities. Furthermore, measurements performed before and after the injury, as well as before, during and at the end of the season [49, 50], may allow for early detection of proprioception disorders, and prevent the damage extending to the passive stabilizers of the shoulder by the implementation of an appropriate rehabilitation procedure [18, 38, 51, 52]. Moreover, the results also suggest that ignoring the time required for passive shoulder stabilizer damage to heal, disregarding its neuroplasticity potential [52], and returning to sporting activities too early do not in fact shorten breaks in career, but extend them. Such activities lead to long-term damage [50, 51] requiring surgical repair, which significantly prolongs these breaks and, in at least 20 % of cases, makes it almost impossible to return to previous levels of overhead sport activity [53].

Our results indicate that differences greater than 15 % for PJPS and greater than 24 % (Table 1) for AJPS for ER and IR can an effective way of identifying shoulders at risk in overhead sport activities. Since differences between 10 to 15 % are acceptable in the case of isokinetic testing [18], and it seems justified to express the limits as percentages rather than degrees as doing so provides a more accurate picture of proprioception. Although the extrapolation of our results to the overhead sport activity population has certain limitations, our findings nevertheless constitute a set of baseline normative PJPS and AJPS values for IR and ER of the shoulder which may be valuable in future studies on arms in the MNP position using an isokinetic dynamometer.

## Conclusions

1. The anatomical structure of the anterior and posterior passive shoulder restraints has no impact on differences in PJPS between ER and IR in a modified neutral shoulder position.
2. The potential for spontaneous healing of anatomical structure of the anterior and posterior passive shoulder restraints influences the recovery of PJPS after arthroscopic release of idiopathic frozen shoulder.
3. Deficits of external rotator peak torque negatively affect AJPS.
4. The use of an isokinetic dynamometer with the arm in the modified neutral shoulder position allows the PJPS and AJPS of ER and IR to be measured with a high level of reproducibility.
5. Differences greater than 15 % for PJPS and 24 % for AJPS of ER and IR of the shoulder can be helpful in future studies as baseline data for selection of particular active and passive shoulder stabilizers at risk.

## Abbreviations

IFS: idiopathic frozen shoulder
PJPS: passive joint position sense
AJPS: active joint position sense
ER: external rotation
IR: internal rotation
I: involved
U: uninvolved
ICC: inter-observer correlation coefficient test
MGHL: middle gleno-humeral ligament
IGHL: inferior gleno-humeral ligament
ABIGHL: anterior band of inferior gleno-humeral ligament
PBIGHL: posterior band of inferior gleno-humeral ligament
